# Towards a Non-pharmacological Intervention on Apathy in Korsakoff’s Syndrome: A Systematic Narrative Review Across Different Clinical Conditions

**DOI:** 10.2147/NDT.S483470

**Published:** 2024-11-13

**Authors:** Maud E G van Dorst, Yvonne C M Rensen, Johanna M H Nijsten, Gwenny T L Janssen, Roy P C Kessels

**Affiliations:** 1 Donders Institute for Brain, Cognition and Behaviour, Radboud University, Nijmegen, the Netherlands; 2 Centre of Excellence for Korsakoff and Alcohol-Related Cognitive Disorders, Vincent van Gogh Institute for Psychiatry, Venray, the Netherlands; 3 Knowledge Centre for Specialized Care, Archipel, Eindhoven, the Netherlands; 4 Department of Primary and Community Care, Radboud Institute for Health Sciences, Radboud University Medical Center, Nijmegen, the Netherlands; 5 Tactus Addiction Care, Deventer, the Netherlands; 6 Klimmendaal Rehabilitation Center, Arnhem, the Netherlands

**Keywords:** non-pharmacological treatment, neuropsychiatric symptoms, alcohol use disorder, dementia, Parkinson’s disease, acquired brain injury

## Abstract

**Abstract:**

Apathy is a quantitative reduction of goal-directed activity, which can be observed in relation to behavior, cognition, emotions and social interaction. It is an invalidating behavioral symptom that is frequently present across different psychiatric conditions and neurocognitive disorders including Korsakoff’s Syndrome (KS). In fact, apathy is one of the most severe behavioral symptoms of KS and has a major impact on the lives of patients and their relatives and other informal caregivers. However, guidelines for the treatment of apathy in KS are currently not available. This systematic narrative review provides a transdiagnostic overview of the effectiveness of different types of non-pharmacological interventions on apathy across different study populations that at symptom-level share characteristics with KS. This evidence may inform the development of an intervention targeting apathy in KS. The included study populations are dementia (due to Alzheimer’s disease, or vascular dementia), Parkinson’s disease, schizophrenia and traumatic brain injury. Through a stepped selection approach and with regard to the Preferred Reporting Items for Systematic Reviews and Meta-Analyses (PRISMA) guidelines, 22 systematic reviews and 32 empirical articles on the non-pharmacological treatment of apathy were identified. The results show a variety of effective non-pharmacological interventions on apathy. In conditions with severe cognitive impairments, successful interventions did not rely on intrinsic motivation, self-monitoring, or illness insight of the patients, but depend on external stimulation and behavioral activation. Since apathy is a multidimensional construct, identification of the extent and type of apathetic behavior before starting an intervention is highly recommended. Furthermore, it is important to adjust the treatment to the patients’ personal interests and needs and embedded in daily care.

**Trial registration:**

CRD42022298464 (PROSPERO).

## Introduction

Chronic and excessive alcohol consumption may result in various degrees of cognitive dysfunction, including deficits in attention, memory, visuospatial functions and executive functions.^1^,^2^ Cognitive functioning may recover after a prolonged period of abstinence,^3^,^4^ but some people continue to show cognitive dysfunction. Korsakoff’s syndrome (KS) is the most severe neuropsychiatric disorder that may occur in the context of chronic and excessive alcohol consumption. KS is a largely irreversible syndrome that may develop in people who suffered from (inadequately treated) Wernicke’s encephalopathy resulting from thiamine depletion. KS is commonly known for its severe memory impairments. However, behavioral symptoms including flattened affect, confabulations, impaired awareness of deficits and apathy are also commonly present in alcoholic KS.^1^,^5^,^6^ Apathy was already being mentioned in the earliest descriptions of the syndrome^7^ and is one of the neuropsychiatric symptoms in KS that have both a high prevalence (ranging from 8%^5^ up to 49.5%^8^) and high severity estimates.^5^

Apathy is a transdiagnostic symptom, as it is frequently present in different psychiatric conditions and neurocognitive disorders, such as Parkinson’s disease, schizophrenia and traumatic brain injury.^9^,^10^ It can be defined as a quantitative reduction of goal-directed activity in comparison to someone’s previous level of functioning and is observed in relation to behavior (eg reduced level of general activity or diminished persistence in maintaining an activity), cognition (eg reduced interests or having difficulty making choices), emotions (eg emotional blunting or affective flattening) and social interaction (eg reduced initiative to or interest in social activities or diminished interest in relatives).^11^ However, overlapping but different definitions of apathy are sometimes used in the literature in different study populations, which may complicate the interpretation of findings and the development of guidelines. Therefore, the International Society for CNS Clinical Trials Methodology Apathy Work Group recently proposed consensus criteria for apathy in the context of neurodegenerative disease, that is, diminished initiative, diminished interest, or diminished emotional expression/responsiveness.^12^

In the field of dementia and traumatic brain injury, apathy is associated with decreased treatment responsiveness or compliance, poorer illness outcome and more problems in activities of daily living, impacting not only the lives of the patients but also those of their families and/or informal caregivers.^13^ In the field of schizophrenia, apathy has been found to disrupt the interaction between the patients and their family members, often contributing to a feeling of rejection in these family members.^14^ Apathy is associated with increased caregiver distress and increased feelings of frustration and misunderstanding, as it can be easily mistaken for laziness, oppositional behavior or emotional distress. Furthermore, apathy is associated with a worse health-related quality of life in nursing-home residents.^15^ A reduction of apathy may thus both improve the patient’s quality of life and reduce the caregiver burden.

Traditionally, research on the treatment of neuropsychiatric symptoms including apathy has focused on pharmacological interventions. Acetylcholinesterase inhibitor (AChI) medications are primarily used for the treatment of neuropsychiatric symptoms in individuals with mild-to-moderate Alzheimer’s’ dementia.^16^,^17^ Also, methylphenidate has been found to be effective in the treatment of apathy in Alzheimer’s dementia.^18^ In schizophrenia, apathy has been successfully treated with antipsychotic medication.^19^,^20^ However, apathy was rarely included as a primary outcome measure in these studies and the severity of neuropsychiatric symptoms at baseline was sometimes very low.^21^ Up until now, the effectiveness of pharmacological interventions for the treatment of apathy is limited.^22^ Furthermore, even though psychotropic medication is often prescribed to treat behavioral symptoms in patients with KS, its use is off label and evidence regarding the effectiveness is lacking.^23^ Non-pharmacological interventions may, however, be more promising and are often recommended as first-line treatment.^24^

Although apathy is reported to be one of the most severe behavioral symptoms of alcoholic KS,^5^ studies on the treatment of apathy in this population are scarce. To our knowledge, only two studies have been performed to date on the treatment of apathy in KS. Results from a pilot study in 38 KS patients showed a decrease on the apathy subscale of the Neuropsychiatric Inventory Questionnaire (NPI-Q) after light intervention therapy in which people with KS were exposed to additional light during the morning hours.^25^ Results from a longitudinal intervention study^26^ did not show a reduction of apathy after implementation of the ABC method (a behavior management technique for use by professional carers). However, both studies have their limitations. For example, both studies approached apathy as a unitary construct, while recent evidence emphasized the multidimensional character of apathy.^27^ More research is thus needed to develop potentially effective interventions for the treatment of apathy as a multidimensional construct in alcoholic KS.

Interventions that have been found to be effective in other populations might guide the development of a new intervention for apathy aimed at patients with KS. The aim of this review is to systematically evaluate the effectiveness of different types of non-pharmacological interventions targeting apathy in different psychiatric conditions and neurocognitive disorders that share characteristics at the symptom level with KS. We will focus on the evidence in dementia, Parkinson’s disease, schizophrenia, and traumatic brain injury. More specifically, people with dementia (due to Alzheimer’s disease or vascular dementia) also have severe memory impairments and problems in daily activities. In addition, individuals with Parkinson’s disease have a comparable prevalence of apathy as well as executive dysfunction. People with schizophrenia share the history of psychiatric symptoms and psychopathology, and also show apathetic behavior and reality-monitoring deficits leading to problems with social functioning and participation.^13^,^28^ Furthermore, the brain abnormalities found in KS share characteristics with other types of acquired brain injury, notably traumatic brain injury, which may also result in a lack of insight and awareness, in addition to apathy.^29^

This systematic review will complement existing literature by providing a transdiagnostic overview. Based on the available evidence in other psychiatric conditions and neurocognitive disorders, we will also present a first proposal on the essential ingredients for an intervention aimed at reducing apathy in individuals with KS, in which to date no studies have been performed on the treatment of apathy.

## Material and Methods

### Search Methods

The Preferred Reporting Items for Systematic Reviews and Meta-Analyses (PRISMA) guidelines were followed to select and explore the literature and report the results in this systematic review. Appropriate MeSH terms were identified with advice from a university librarian. A systematic search was initially conducted on January 1, 2022, and was updated on June 1, 2024, to identify all potentially relevant literature using the Cochrane Library for meta-analyses and systematic reviews, PsycInfo, Medline and Web of Science. Records were selected if they contained the following search terms (or truncated versions): i) “apathy” or “avolition”, and ii) “non-pharmacological treatment”, “intervention”, “therapy” or “training”. Furthermore, these systematic searches were conducted for each study population separately using the following search terms (or truncated versions): “dementia”, “Alzheimer”s disease’, “Parkinson”s disease’, “Schizophrenia” or “traumatic brain injury”. Due to large differences of available literature in the different target populations, we used a stepped selection approach. First, we searched for systematic reviews. Next, if systematic reviews were not available or were published before 2021, we continued searching for individual empirical studies. When a systematic review was available but not up to date, we searched for individual studies published after the conclusion of the search in that review.

### Inclusion Criteria

All reviews and empirical articles were screened, assessed and selected independently by the first two authors. No automation tools were used. Selection differences were discussed during consensus meetings. The included reviews and articles met the following three inclusion criteria:

1. The study samples comprised people with a diagnosis of Alzheimer’s disease, vascular dementia, Parkinson’s disease, schizophrenia or traumatic brain injury. Either studies in ambulatory or in clinical patients could be included;
2. Apathy or a closely related symptom (eg avolition, social amotivation, social withdrawal, goal-oriented thinking, self-initiation and physical or social activity) was measured quantitatively with a patient-report or informant-report version of assessment tools (questionnaires or observation scales, such as the Apathy Evaluation Scale (AES),^30^ the Apathy Scale (AS),^31^ the Lille Apathy Rating Scale (LARS)^32^ and the Neuropsychiatric Inventory (NPI)^33^);
3. The studies included a non-pharmacological intervention;
4. The studies had to be published in English, Dutch or German.

### Data Processing

Given the large expected variability in methodology and outcome measures across studies, we could not perform a formal quantitative meta-analysis. For each included study population and for each included record, the first two authors reviewed the findings independently in a narrative manner, discussing the findings for each specific type of intervention. Next, they outlined the effectiveness of each type of intervention or technique visually in tables using a plus sign indicating a positive effect on the reduction of apathy (or a minus sign for a negative effect). An equal sign was used if no proven reduction of apathy was found after an intervention. A combination of different signs indicates mixed evidence for the effectiveness of this type of intervention. All included types of intervention including abbreviations and brief explanations are included in Table 1.

**Table 1:** Descriptions and Abbreviations of the Types of Interventions Referred to in the Current Systematic Review

Type of intervention	Abbreviation	Description
Animal-assisted therapy	AAT	This therapy is provided in different forms, but always includes the presence of an animal.
Art therapy	AT	Therapy that involves creative processes and activities to improve impaired functioning and/or someone’s wellbeing.
Behavioral activation	BA	Behavioral activation (in some studies referred to as activity scheduling) is a technique that helps patients to perform their desired activities by scheduling, structuring and monitoring the activities.
Cognitive Behavioral Therapy	CBT	Therapy in which thoughts, feelings and behavior are analyzed in order to make adjustments to unhelpful thoughts and behavior. One example of an included CBT is the Positive Emotion Program for Schizophrenia (PEPS).
Cognitive stimulation	CS	Providing stimuli or activities that may trigger a response. Examples are: reality orientation, playing games and providing themed material or “activity kits” that trigger associations and/or actions.
Compensatory cognitive strategy training	CCST	This intervention provides and trains the use of strategies to support learning/memory, attention and other cognitive functions.
External cueing	EC	Providing cues or stimuli in the environment, for example reminder alerts.
Goal Management Training	GMT	Goal Management Training includes problem orientation and definition, setting goals, generating activities that are needed to reach these goals and maintain the problem-solving process on a meta-cognitive level. In this review, we also included closely related interventions like problem solving training (PST). Some articles focused particularly on activity scheduling and monitoring (components of GMT), those interventions are in de current review referred to as behavioral activation (BA).
Guided activities	GA	Performing (personalized) therapeutic, leisure or daily activities with guidance.
Imagery therapy	IT	A therapeutic technique that uses imagined scenes by patients themselves.
Motivational Interviewing	MI	Motivational Interviewing is a verbal communication technique used to facilitate or trigger a change in behavior by exploring someone’s ambivalence.
Multi-sensory stimulation	MMS	Multi-sensory therapy appealing to more than one sense. This therapy usually consists of a combination of visual, auditory and tactile stimuli and is most often conducted in specially designed rooms with a variety of lights and gentle music.
Music therapy	MT	Music therapy is an evidence-based therapy that may involve playing a musical instrument, listening to music or using music to exchange information.
Personal attention	PA	This intervention method provides one-on-one time in which there is room for personal conversations and/or attention to someone’s emotions. Social support is a closely related term.
Positive reinforcement	PR	An intervention method that involves rewarding of desired behavior.
Physical exercise	PhE	Interventions that require physical effort or engaging in physical activities.
Psychoeducation	PsE	This intervention method consists of providing information.
Psychomotor therapy	PMT	This therapy involves physical exercises in order to increase body awareness and physical activity.
Social robot	SR	This therapy is provided in different forms, but always includes the presence of a social robot that can interact with people through different kinds of sensors and that is programmable.
Social skill training	SST	This training includes interactive exercises to train social communication and recognition of facial expressions.
Training nursing staff	TNS	This technique is used to influence the patients’ behavior, well-being and/or quality of life by giving instructions to professional caregivers.
Virtual reality	VR	A computer-generated technique that creates 3D scenes and objects.
Validation therapy	VT	A technique of therapeutic communication in which the emotional aspect of a conversation is more important than the factual content. This technique sometimes involves validation of untruths.

## Results

Below we present the results of the search on non-pharmacological interventions on apathy in patients with dementia, Parkinson’s disease (PD), schizophrenia and traumatic brain injury (TBI). Interventions that have found to be effective in these populations might guide the development of an intervention for apathy in patients with KS.

### Dementia

#### Search Results

**Figure 1:**
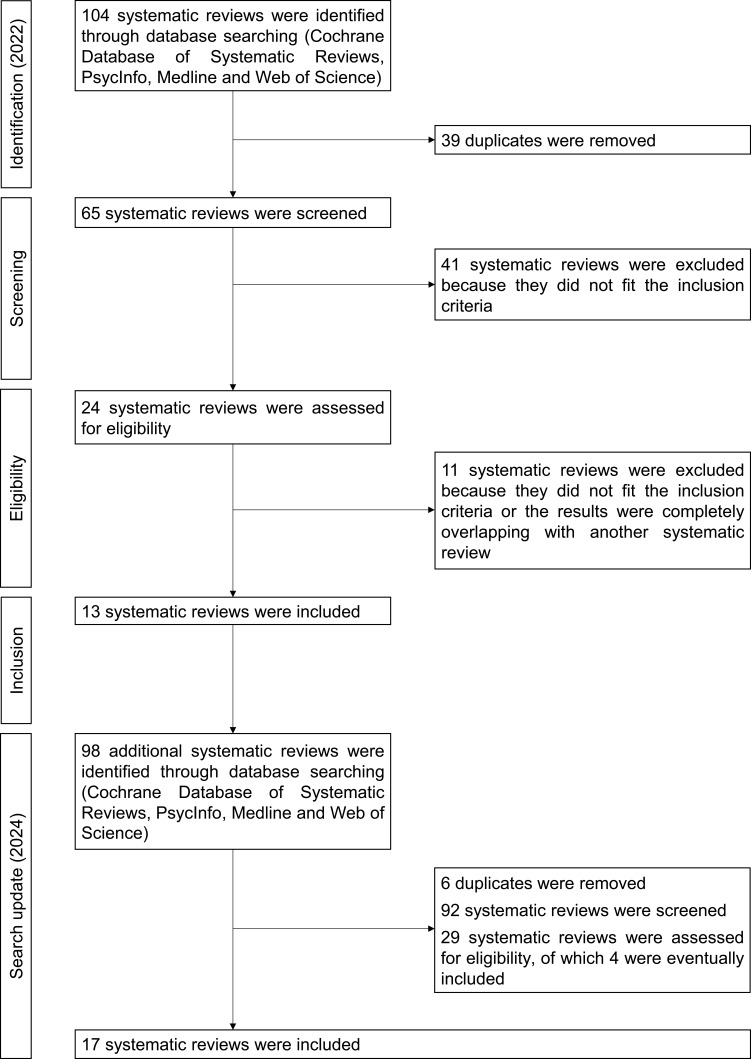
Flowchart illustrating the phases of study selection in the field of dementia.

Seventeen systematic reviews were included. The search outcome and selection process are presented in a flowchart in Figure 1. The types of interventions described in the included systematic reviews and their efficacy are presented in Table 2 and outlined below.

**Table 2:** Types of Intervention and Their Efficacy in Targeting Apathy in People with Dementia, as Described in the Included Systematic Reviews

Authors and year of publication	k	Type of intervention													
		MSS	VT	CS	MT	PA	AT	TNS	GA	PhE	AAT	PhE + CS	MT + AT or PMT	SR	VR
Verkaik et al 2005^34^	10	+	=	=											
Fisher-Terworth et al 2009^35^	27	+			+										
Lane-Brown & Tate 2009^36^	19	+/=			+	=	+	=	+						
Brodaty et al 2012^37^	13	=	=		+	=		=	+	=					
Bernabei et al 2013^38^	1										+				
De Oliveira et al 2015^39^	1				+										
Fukushima et al 2016^40^	2						+					+			
Goris et al 2016^41^	16	+		+	+	+	+	+/=	+				+	=	
Theleritis et al 2018^42^	43	+		+/=	+	+/=	+	+/=	+	+/=		+	+	+/=	
Tsoi et al 2018^43^	3				+										
Zafra-Tanaka et al 2019^44^	1										+				
Lam et al 2020^45^	3				+										
Lin et al 2020^46^	1						+								
Chan et al 2022^47^	3			+										+	+
Ho et al 2022^48^	6														+
Ardelean & Redolat 2024^49^	6			=	+									+	
Peng et al 2024^50^	3													+	

**Notes**: k: number of included studies that are relevant for the current review; +: significant evidence for reduction of apathy; =: no significant evidence for reduction of apathy.

**Abbreviations**: MSS, multi-sensory stimulation; VT, validation therapy, CS, cognitive stimulation; MT, music therapy; PA, personal attention; AT, art therapy; TNS, training for nursing staff; GA, guided activities; PhE, physical exercise; AAT, animal assisted therapy, PMT, psycho-motor therapy; SR, social robot; VR, virtual reality.

#### Music Therapy

Music therapy (MT) is the most extensively studied intervention, with exclusively positive results with respect to its effectiveness. Eight reviews presented positive effects showing reduced apathy and enhanced engagement in patients with different stages of dementia.^35–37^,^39^,^41–43^,^45^,^49^ Live music sessions, including interactive MT using instruments and listening to live music, were found to reduce apathy more than listening to pre-recorded music, silent periods, or an active control intervention.^36^,^37^,^39^,^41^,^43^ However, a reduction of apathy was also reported after receptive MT.^43^,^49^ All included reviews demonstrated short-term effects on apathy. Two reviews included results of follow-up measurements and both described that the effect of MT did not persist after the end of the intervention.^35^,^37^

#### Art Therapy

Five reviews presented the effects of art therapy (AT) on apathy, all with positive effects.^36^,^40–42^,^46^ All reviewed interventions consisted of multiple sessions with coloring and drawing as primary task, mostly done on a weekly basis. In one review, AT was compared to a control intervention with calculation exercises and also adjusted for depressive symptoms, resulting in a significantly larger reduction of apathy after AT.^46^

#### Multi-sensory Stimulation

Four reviews showed positive effects of multi-sensory stimulation (MMS) on apathy,^34^,^35^,^41^,^42^ with one review showing a significantly larger reduction of apathy after MMS compared to activity therapy or a stay in the living room.^34^ Some of the articles included in Verkaik et al^34^ specified the reduction of apathy as “improved attentiveness to the environment”, which is a characteristic description of apathy for this type of study. One review presented mixed evidence for MMS^36^ and one review did not show any beneficial effect of MMS on apathy^37^ compared to attention-only or activity control groups. All reviews only included people with moderate-to-severe or severe dementia. As in the field of MT, most studies examined only short-term effects of MSS or concluded that positive effects were not maintained over longer time periods after the intervention was ended.

#### Activities with Guidance

Four reviews showed beneficial effects on apathy of a broad group of personalized and often individually tailored therapeutic activities that included guidance, activity engagement and the ability to keep busy in dementia (guided activities; GA).^36^,^37^,^41^,^42^ Personalizing activities to the preferences of the patients enhanced this effect more than matching interventions to skill level.^41^,^42^ For example, Simulated Presence (personalized audiotapes) in patients with Alzheimer’s disease decreased apathy (ie, level of interest, withdrawn behavior) more than placebo and usual care.^51^ Moreover, a double-blind randomized controlled trial examined the efficacy of activities customized to the functional level of cognitively impaired residents or their personal preference. This study reported that taking into account the personal preference of the patients was the critical component that produced the most effective results for improving apathy (ie, engagement, capturing attention, increasing alertness).^52^

#### Social Robots

Interventions with social robots (SR) are currently getting increased attention. The search update conducted in June 2024 yielded exclusively positive results of interventions with SR on apathy levels in people with dementia.^47^,^49^,^50^ In the reviewed articles, both pet-robots and human-like doll robots were used.

#### Personal Attention

Mixed evidence was found for the efficacy of personal attention (PA), provided in one-on-one time where there is room for personal conversations and attention to one’s emotions. Two systematic reviews showed no beneficial effects of PA,^36^,^37^ one review yielded mixed results^42^ and one review showed reduce apathy after PA.^41^

#### Other Interventions

Two reviews showed mixed evidence for interventions targeting apathy indirectly by training nursing staff and two other reviews did not find any positive evidence for such interventions.^36^,^37^,^41^,^42^ An emotion-oriented care training for nursing staff yielded no reductions of apathy compared to usual care.^36^,^37^ However, concrete staff instructions with dos and don’ts were found to reduce apathy.^41^,^42^ There is no convincing evidence for beneficial effects of singular validation therapy, cognitive stimulation or physical exercise on apathy or related constructs like “withdrawn behavior”. However, two reviews presented beneficial effects on apathy of a 12-week combined intervention with daily sessions (5 days a week) with one hour of physical activity followed by one hour of cognitive stimulation (ie activities to enhance orientation, memory, or executive skills).^40^,^42^ A significant decrease in apathy was also seen after music therapy combined with either art therapy or psychomotor therapy. Interventions using social robots give mixed evidence in reducing apathy. Positive effects were only present in patients living in nursing homes, but not in patients visiting day care centres.^41^,^42^ Results of animal-assisted therapy (AAT) are inconclusive but promising. The three included systematic reviews on this type of intervention presented positive results of AAT on reducing apathy. Finally, one systematic review^48^ and one article reviewed in Chan et al^47^ showed beneficial effects of virtual reality interventions on apathy.

#### Summary of Results for Dementia

In the field of dementia, music therapy, art therapy, multi-sensory stimulation, guided activities and social robots were reported most frequently as being effective to treat apathy.^44–51^,^53–55^ Combined interventions with physical exercise and cognitive stimulation, as well as animal-assisted therapy and virtual reality have been studied less often, but have been proven successful.^47^,^53^,^56^,^57^ Mixed evidence was found for isolated cognitive stimulation, personal attention, training nursing staff and physical exercise.^34–37^,^39–46^−^53^ There is no scientific evidence for validation therapy as an effective intervention to reduce apathy in dementia.^34^,^37^

### Parkinson’s Disease

#### Search Results

**Figure 2:**
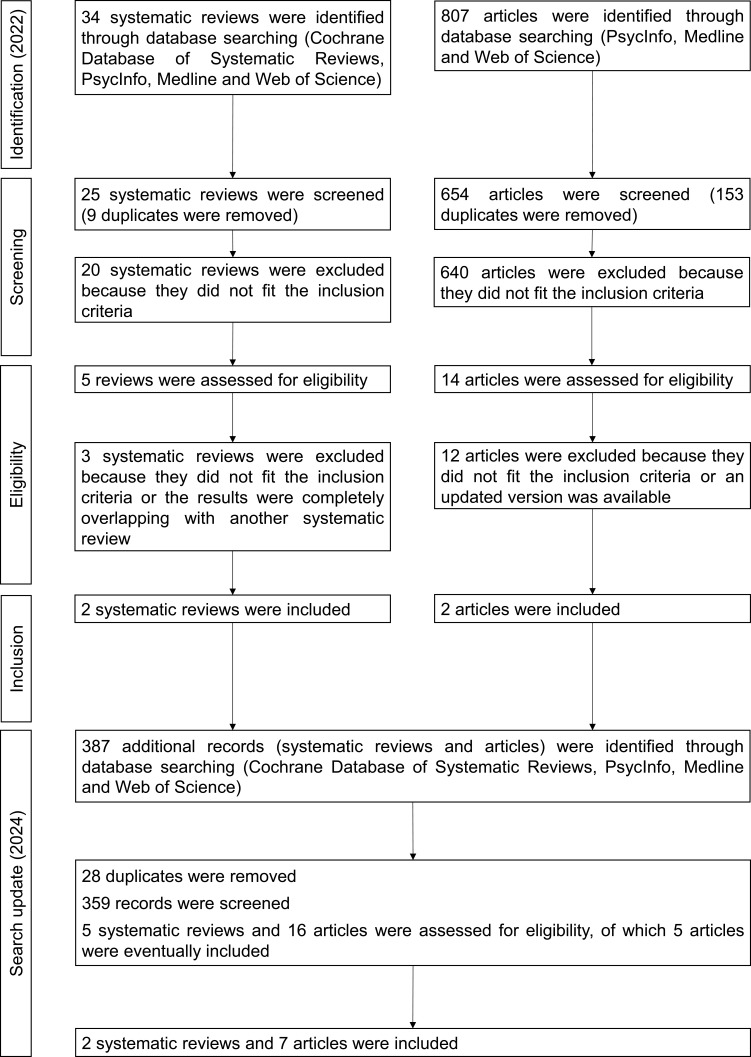
Flowchart illustrating the phases of study selection in the field of Parkinson’s disease.

Two systematic reviews were included. However, because the focus in these reviews was mainly on the effect of physical exercise on apathy, an additional search for individual, empirical articles was conducted and led (including the results of the search update) to the inclusion of seven empirical articles. The selection process is presented in a flowchart in Figure 2. The types of intervention as presented in the included reviews and empirical articles and their efficacy are presented in Table 3.

**Table 3:** Types of Intervention and Their Efficacy in Targeting Apathy in People with Parkinson’s Disease, as Described in the Included Systematic Reviews and Articles

	k/n	PhE				BA	CCST	CBT	MT
		Nordic Walking	Fitness exercises	Dance therapy	Boxing				
Cusso et al 2016^53^	k=3	+	+/=	=					
Zhang et al 2019^54^	k=2			=					
Larson et al 2022^55^	n=1709				=				
Patel et al 2023^56^	n=22				=				
Butterfield et al 2017^57^	n=34					+			
Diez-Cirarda et al 2017^58^	n=15						=		
Piers et al 2022^59^	n=1							+	
Piers et al 2023^60^	n=12							+	
Shah-Zamora et al 2024^61^	n=16								+

**Notes**: k: number of included studies that are relevant for the current review; n: number of the included participants; +: significant evidence for reduction of apathy; =: no significant evidence for reduction of apathy.

**Abbreviations**: PhE, physical exercise; BA, behavioral activation; CCST, compensatory cognitive strategy training; CBT, Cognitive Behavioral Therapy; MT, music therapy.

#### Physical Exercise

Mixed evidence was found for the effectiveness of physical exercise interventions on apathy in PD. One systematic review showed reduced apathy after a 12-week Nordic Walking intervention with two 60-minute sessions a week compared to a control group performing no exercise.^53^ This systematic review also showed beneficial effects of an individualized fitness exercise program on apathy. However, this reduction was not larger compared to the effects of a group exercise program or performing fitness exercises at home.^53^ Two systematic reviews did not show a significant reduction of apathy after different dance interventions.^53^,^54^ One of the excluded reviews (excluded because apathy was not measured explicitly) did, however, describe a beneficial effect of dance intervention on social interaction and communication. Due to the lack of information about how social interaction was measured, it remains unclear if this may be seen as an effect on (social) apathy.^62^ Boxing interventions did not enhance reduction of apathy in PD.^55^,^56^

#### Other Interventions

One empirical study investigated behavioral activation in the form of a telephone-based 6-week intervention providing activity scheduling and monitoring in 34 people with PD and found a significant reduction of apathy after the intervention.^57^ This effect was maintained at 1-month follow-up. In another empirical study, a group-based compensatory cognitive strategy training did not reduce apathy in 15 people with PD. The intervention included three 1-hour sessions a week for 13 weeks.^58^ One case study and one pilot study showed beneficial effects of Cognitive Behavioral Therapy on apathy.^59^,^60^ One RCT showed a reduction of apathy after virtual music therapy, but caregiver burden did not change after the music therapy.^61^

#### Summary of Results for Parkinson’s Disease

The two systematic reviews on non-pharmacological interventions targeting apathy in the field of PD focused exclusively on physical exercise.^53^,^54^ Both reviews concluded that some specific physical exercise interventions, but not all, are effective in reducing apathy. Furthermore, limited but positive evidence was found for Cognitive Behavioral Therapy, behavioral activation (activity scheduling and monitoring on apathy) and music therapy.^57^,^59–61^ One article did not show a significant reduction of apathy after compensatory cognitive strategy training.^58^

### Schizophrenia

#### Search Results

**Figure 3:**
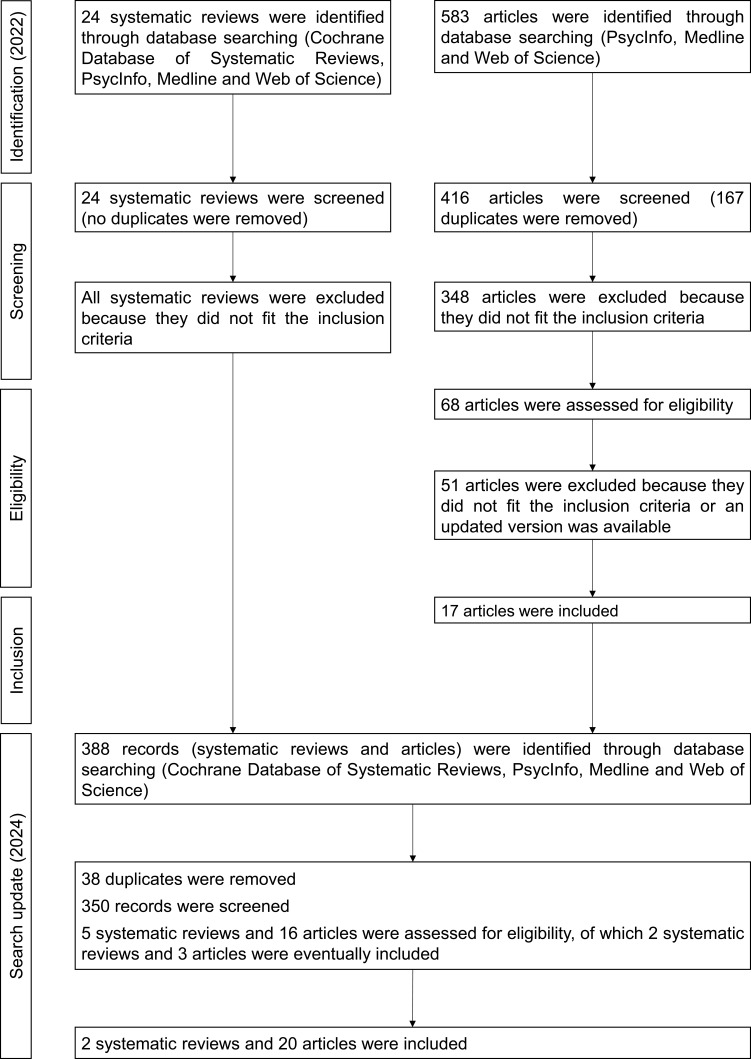
Flowchart illustrating the phases of study selection in the field of schizophrenia.

Two systematic reviews and twenty empirical articles were included. The flowchart in Figure 3 shows the selection process. The types of intervention conducted in this populations and their efficacy are presented in Table 4.

**Table 4:** Types of Intervention and Their Efficacy in Targeting Apathy in People with Schizophrenia, as Described in the Included Systematic Reviews and Articles

Authors and year of publication	k/n	Type of intervention								
		PMT	PhE	MT	CBT	SST	CCST	PR	GMT	SST + PsE
Isabelinha et al 2023^63^	k=4	+	=	=						
Lyngstad et al 2023^64^	k=9				+	+	+			
Skelly & Haslerud 1952^65^	n=39			+						
Schaefer & Martin 1966^66^	n=20							+		
Johns 2002^67^	n=4				+ (group)					
Torres et al 2002^68^	n=49					+				
Staring et al 2013^69^	n=21				+ (individual)					
Williams et al 2014^70^	n=27				+ (group)					
Favrod et al 2015^71^	n=31				+					
Grant et al 2017^72^	n=60				+ (group)					
Palumbo et al 2017^73^	n=5					+				
González-Pando et al 2018^74^	n=9					=		=		
Favrod et al 2019^75^	n=80				=					
Favrod et al 2019^76^	n=21				+					
Ventura et al 2019^77^	n=80						+			
Mahmood et al 2019^78^	n=43						+			
Thonon et al 2020^79^	n=3								+ (short version)	
Thonon et al 2020^80^	n=8								+ (full version)	
Austin et al 2021^81^	n=51									+
Chai et al 2023^82^	n=100									+
Emami et al 2023^83^	n=64						+			
Oh et al 2023^84^	n=70						=			

**Notes**: n: number of the included participants; +: significant evidence for reduction of apathy; =: no significant evidence for reduction of apathy.

**Abbreviations**: PMT, psychomotor therapy; PhE, physical exercise; MT, music therapy; CBT, Cognitive Behavioral Therapy; SST, social skill training; CCST, compensatory cognitive strategy training; PR, positive reinforcement; GMT, Goal Management Training; PsE, psychoeducation.

#### Cognitive Behavioral Therapy

One systematic review and seven empirical articles presented the effects of Cognitive Behavioral Therapy (CBT) and their results are mainly positive.^64^,^67^,^69–72^,^75^,^76^ Both individual and group CBT proved effective in reducing apathy compared to treatment as usual. In a study of Grant et al,^72^ effects of CBT persisted until over a year after the intervention. Two non-randomized empirical studies on the “positive emotion program for schizophrenia” (PEPS), a specific type of CBT, showed positive effects on apathy after the intervention^71^,^76^ and one empirical study yielded no greater reduction of apathy immediately after this type of CBT compared to treatment as usual (TAU).^75^ However, significant reductions of apathy were observed six months after the CBT and were significantly larger compared to TAU. Although results of CBT are mainly positive, one study showed that despite the reduction of apathy based on observer-ratings, the participants’ subjective experience of apathy did not change after the intervention.^67^

#### Compensatory Cognitive Strategy Training

One systematic review and three empirical studies showed positive results on apathy after interventions providing compensatory cognitive strategy training (CCST) to support for instance learning/memory, attention or executive functioning.^64^,^77^,^78^,^83^ A 12-week group based CCST combined with pharmacotherapy reduced social amotivation more than pharmacotherapy only.^78^ Computerized CCST proved effective as well. Completing this training resulted in a significant greater reduction of apathy compared to completing an online lifestyle training focusing on healthy behavior.^77^ Largest improvements in apathy in both studies were seen in combination with the use of medication.

#### Goal Management Training

Two empirical studies showed a positive effect of Goal Management Training on the reduction of apathy.^79^,^80^ Both a version consisting of 30 individual session over 12 months and a shorter version consisting of eight individual sessions over two months yielded positive results. These effects were seen both in individuals with mild and in those with severe levels of apathy at baseline.

#### Social Skill Training

Positive results of social skill training (SST) are described in one systematic review.^64^ Furthermore, SST yielded positive results in three out of four empirical studies. Two studies showed a reduction of apathy after SST^68^,^73^ and one study in which SST was combined with psychoeducation also yielded beneficial effects on the reduction of apathy.^81^ One empirical study showed no beneficial effects of SST on the reduction of apathy.^74^

#### Other Interventions

One review showed a positive effect of a body-oriented psychotherapy on apathy^63^ and one review showed a positive effect of a yoga intervention on apathy.^64^ One study found that listening to music reduced apathy.^65^ A positive reinforcement intervention with social behavior being rewarded by trained nursing staff did not reduce apathy.^74^

#### Summary of Results for Schizophrenia

In the field of schizophrenia and schizoaffective disorders, Cognitive Behavioral Therapy is most often reported as being effective in reducing apathy. Compensatory cognitive strategy training, Goal Management Training, social skill training and music therapy have been studied less often, but the majority of the included studies show positive effects on the reduction of apathy. Positive reinforcement interventions were not found to be effective in reducing apathy. In all, most interventions on apathy in the field of schizophrenia require insight and awareness of one’s own behavior, and the ability to monitor and evaluate this behavior is needed to make the interventions succeed.

### Traumatic Brain Injury

#### Search Results

**Figure 4:**
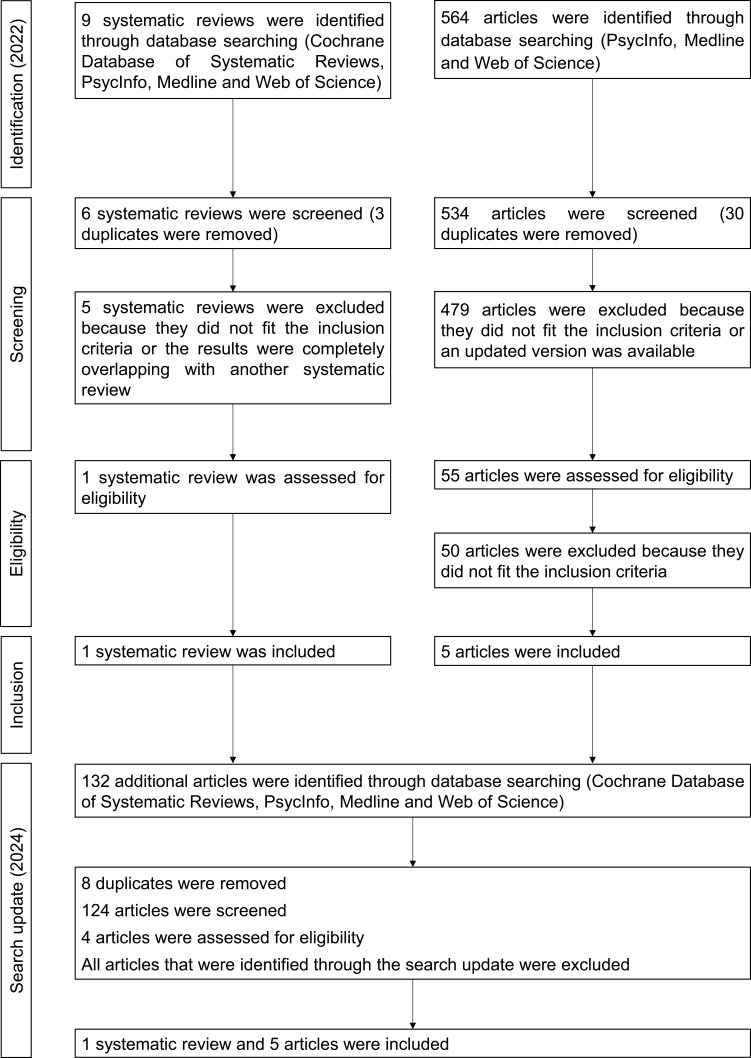
Flowchart illustrating the phases of study selection in the field of traumatic brain injury.

One systematic review and five empirical articles were included via the stepped approach selection. The selection process is presented in a flowchart in Figure 4. The types of intervention examined in patients with TBI and their efficacy are presented in Table 5.

**Table 5:** Types of Intervention and Their Efficacy in Targeting Apathy in People with Traumatic Brain Injury, as Described in the Included Systematic Review and Articles

Authors and year of publication	k/n	Type of intervention							
		GMT	BA	EC	CCST	MI	GA	PsE	IT
Lane-Brown & Tate 2009^36^	k=6	+	+	+	+/=				
Lane-Brown& Tate 2010^85^	n=1		+			+			
Mochizuki-Kawai et al 2018^86^	n=27						=		
Campbell et al 2019^87^	n=24							+	=
Tate et al 2020^88^	n=7	+							
Gertler & Tate 2021^89^	n=2	=							

**Notes**: k: number of included studies that are relevant for the current review; n: number of the included participants; +: significant evidence for reduction of apathy; =: no significant evidence for reduction of apathy.

**Abbreviations**: GMT, Goal Management Training; BA, behavioral activation; EC, external cuing; CCST, compensatory cognitive strategy training; MI, Motivational Interviewing; GA, guided activities; PsE, psychoeducation; IT, imagery therapy.

#### Goal Management Training

Two out of three articles on Goal Management Training (GMT) yielded positive results on the reduction of apathy. One systematic review showed a significant increase in goal-oriented thinking in patients with TBI after GMT.^38^ This beneficial effect was greater compared to a memory training. One empirical study presented increased meaningful activities after GMT in a study sample of seven patients with severe TBI.^88^ One notable aspect is that participants included in this study were motivated to increase their level of activity and participation. The results of another empirical study did not show increased physical or social activity due to individual GMT.^89^

#### Behavioral Activation and Concurrent Intervention Methods

Five single-case studies reviewed in the included systematic review and an included empirical article from that review showed improved self-initiation after behavioral activation interventions with checklists, reminder alerts, external cues or activity scheduling in individuals with TBI based on self- and caregiver report.^38^,^85^ The persistence of the effects of these interventions varies greatly between individuals. In one of these single case studies, the intervention included also Motivational Interviewing.^85^

#### Other Interventions

Two single-case studies from the included systematic review showed mixed evidence for compensatory cognitive strategy training (CCST).^36^ CCST was found to be effective in reducing apathy when it was presented in a paper-and-pencil version, not in a computerized version. Both versions were given in daily one-hour sessions during three weeks. One study showed no reductions of apathy after a guided group activity (ie a structured floral arrangement intervention), nor after TAU in a day-care facility.^86^ The implemented activity in this study was the same for all participants and was not tailored to individual preferences or interests. One empirical study examined the effects of compassion focused imagery therapy (IT) in 24 patients with TBI.^87^ Before the imagery intervention, participants watched a preparatory video in which a psychologist provided psychoeducation to an actor by explaining and demonstrating IT. The motivation for the intervention increased significantly after watching this educational video, but IT on itself did not yield an effect on apathy.^87^

#### Summary of Results for TBI

Based on the summarized results of one systematic review and five empirical articles on apathy in TBI, most positive evidence was found for Goal Management Training and behavioral activation combined with structuring techniques and/or Motivational Interviewing. Although studies on these types of interventions only used single-case designs or very small samples, results were mostly positive. Promising yet inconclusive evidence was found for psychoeducation and compensatory cognitive strategy training in analogue version. Guided activities in groups, not tailored to personal interests or skills, and imagery therapy yielded no reduction of apathy.

## Discussion

The aim of the current review was to provide a transdiagnostic overview of the non-pharmacological treatment of apathy in study populations with overlapping characteristics to Korsakoff’s syndrome (KS). Our search identified 22 systematic reviews and 32 empirical articles published in the last seven decades across four different study populations, including dementia (due to Alzheimer’s disease (AD) or vascular dementia (VD)), Parkinson’s disease (PD), schizophrenia and traumatic brain injury (TBI). The results of the current review may help to identify essential ingredients for developing a non-pharmacological treatment targeting apathy in patients with KS.

In the field of dementia, convincing evidence was found for the efficacy of music therapy, art therapy, multi-sensory stimulation, personalized guided activities and social robots.^34–37^,^39–43^,^45–47^,^49^,^50^ Combined interventions with physical exercise and cognitive stimulation, as well as animal-assisted therapy and virtual reality interventions are promising but have been studied less often in patients with dementia.^38^,^40^,^42^,^44^,^47^,^48^ In the field of PD, some specific physical exercise interventions, behavioral activation, Cognitive Behavioral Therapy and music therapy were found to reduce apathy.^53^,^54^,^57^,^59–61^ However, results on the non-pharmacological treatment of apathy in the field of PD are less conclusive compared to the other included populations. In the field of schizophrenia, convincing evidence was found for Cognitive Behavioral Therapy.^67^,^69–72^,^75^,^76^ Furthermore, compensatory cognitive strategy training, Goal Management Training, social skill training and music therapy have been studied less often, but the majority of the included studies showed positive effects on apathy in people with schizophrenia.^65^,^68^,^73^,^77–81^ In the field of TBI, positive evidence was found for Goal Management Training and behavioral activation combined with structuring techniques and/or Motivational Interviewing.^36^,^85^,^88^ Psychoeducation, external cuing and Motivational Interviewing were also found to reduce apathy.^36^,^85^,^87^ Additionally, our findings show that interventions for the different populations differed with respect to their focus (that is, some focus on techniques that externally stimulate the participants’ senses or manipulate the environment, but other interventions apply techniques that activate internal processes and/or require meta-cognitive thinking). For instance, in the field of dementia, PD, and TBI, most successful interventions did not rely on active participation (or even motivation) from the patients themselves, whereas successful interventions in the field of schizophrenia required active participation and/or an intrinsic motivation to reduce apathetic behavior.^34–36^,^67^,^69^,^88^ Differences in treatment approaches may also be the result of the apathy characteristics of specific populations, for instance, in relation to the multifaceted nature of the concept apathy or the severity of the apathy. For example, apathy in schizophrenia is considered the result of a diminished capacity to anticipate pleasant experiences or the achievement of goals.^90^ However, in dementia and TBI, lack of illness insight and awareness may be an important characteristic of apathy. Since lack of insight and awareness is also prominent in individuals with KS, the use of meta-cognitive techniques or techniques that require intrinsic motivation to change behavior are not to be recommended for this population.

A point of discussion is whether interventions should be personalized or not in order to reduce apathy. In the field of dementia, a greater reduction of apathy has been reported following personalized interventions compared with placebo, care as usual or non-personalized interventions.^51^,^52^ In the field of TBI, non-personalized interventions with guided activities did not reduce apathy while a personalized intervention with guided activities did reduce apathy. In all included populations, we reviewed effective intervention methods that naturally have a personalized character, like Goal Management Training and Cognitive Behavioral Therapy. This suggests that, in general, any type of intervention might reduce apathy compared with no intervention. However, personalized interventions might especially be effective in reducing apathy. It might be beneficial to personalize and tailor interventions in such a way that they meet the patients’ own desired goals, interests, or preferences. Tailoring and personalizing interventions is in agreement with the recommendations from researchers and healthcare professionals working in the field of brain disorders and apathy, published in 2020.^91^

As illustrated by the current findings, research on non-pharmacological treatment of apathy has a long tradition (with the first studies in our search being published in 1952). Although this large body of evidence is informative, it also comes with limitations. That is, definitions of apathy and its measurement have changed over the last decades. Moreover, multiple overlapping but slightly contradicting definitions of apathy are used in different populations in the modern literature. As a result, apathy may be approached differently in different study populations. Besides, some studies included in this review labelled closely related, yet distinct constructs like “the level of physical activity”, “involvement in daily activities”, “social amotivation”, “withdrawn behavior” or “attentiveness to the environment” – sometimes assessed by unvalidated rating scales – as apathy.^34^,^38^,^51^,^88^,^89^ The variety of instruments that have been used to measure apathy is also a clear limitation of research in this field. Moreover, most instruments approach apathy as one unitary construct or measure only one specific dimension of apathy. However, apathy is argued to be a multidimensional construct.^11^,^27^ Our current findings illustrate that valid assessment of apathy in all its forms is an important precondition for finding suitable treatment.^91^,^92^

For all identified successful interventions, a disadvantage may be that training specific activities may only lead to an increase of that specific activity, thus leaving apathy or lack of motivation for other activities unaffected. For example, in an included review of Verkaik et al,^34^ both multi-sensory stimulation and reality orientation reduced apathy. However, people who participated in the multi-sensory stimulation study showed increased attentiveness to their environment and people who participated in the reality orientation study showed decreased withdrawn behavior. The increase in both outcome measures was interpreted as a reduction of apathy.

The majority of the studies reviewed did not include follow-up assessments of apathy. Those studies that included follow-up assessments demonstrated that apathy ratings often returned to baseline at longer-term follow-up assessments.^34^,^35^,^37^,^41^,^42^ Finally, it should be noted that the research methods of all included reviews and empirical articles vary greatly, making a formal meta-analysis using standardized effect sizes not possible. Also, many of the studies discussed in this review have been performed in small samples or adopted single-case designs that warrant replication in larger samples.

### Transdiagnostic Conclusion and Clinical Recommendations

This review aims to contribute to the development of a non-pharmacological intervention on apathy in KS. Although specific interventions in treating apathy in other psychiatric conditions and neurocognitive disorders vary considerably, we also identified important transdiagnostic communalities. That is, in conditions with severe cognitive impairments, effective non-pharmacological interventions for apathy should not rely on intrinsic motivation, self-monitoring or illness insight of the patients, but should rely on external stimulation and behavioral activation. Furthermore, effective interventions across conditions are typically personalized in nature. Finally, any type of intervention to reduce apathetic behavior is preferred over no intervention at all. Based on these transdiagnostic results, we present a first proposal highlighting possible effective ingredients for an intervention aimed at reducing apathy in individuals with KS.

With respect to the often severe levels of apathy and the lack of illness insight in patients with KS, it is recommended that interventions should clearly not rely on self-initiative from the patients or meta-cognitive thinking. Based on these specific characteristics of KS and the current results from this broad summary, we recommend an intervention that unconsciously encourages activation by external stimulation and/or manipulation of the patient’ s environment. Furthermore, tailoring interventions to the specific interests or needs of the KS patient are highly recommended. Examples of specific methods that comply with these recommendations and have been proven successful in different patient populations include music therapy, art therapy, (multi-)sensory stimulation, personalized activities with guidance and techniques that externally stimulate behavior such as external cues or a structured environment.

Since apathy is a multidimensional construct, identification of the extent and dimension of apathy in the individual patient is recommended before the start of the intervention. It should also be stressed that for interventions to be successful in clinical practice, they should be embedded well in daily care, making sure that daily caregivers are familiarized with the intervention protocol.^26^ An example of a specific intervention that meets all the above criteria has been recently described for dementia,^93^ that is, the Shared Action for Breaking through Apathy program (SABA). This intervention could be modified for use in the KS population, but its efficacy and effectiveness should be studied empirically in future research.
